# Antibacterial activity of peptaibols from *Trichoderma longibrachiatum* SMF2 against gram-negative *Xanthomonas oryzae* pv. *oryzae*, the causal agent of bacterial leaf blight on rice

**DOI:** 10.3389/fmicb.2022.1034779

**Published:** 2022-10-11

**Authors:** Yu-Qiang Zhang, Shan Zhang, Mei-Ling Sun, Hai-Nan Su, Hao-Yang Li, Kun-Liu, Yu-Zhong Zhang, Xiu-Lan Chen, Hai-Yan Cao, Xiao-Yan Song

**Affiliations:** ^1^State Key Laboratory of Microbial Technology, Marine Biotechnology Research Center, Shandong University, Qingdao, China; ^2^College of Marine Life Sciences & Frontiers Science Center for Deep Ocean Multispheres and Earth System, Ocean University of China, Qingdao, China; ^3^Laboratory for Marine Biology and Biotechnology, Pilot National Laboratory for Marine Science and Technology, Qingdao, China

**Keywords:** *Trichoderma longibrachiatum* SMF2, Trichokonins A, *Xanthomonas oryzae* pv. *oryzae*, biological control, bacterial leaf blight (BLB)

## Abstract

Bacterial leaf blight caused by Gram-negative pathogen *Xanthomonas oryzae* pv. *oryzae* (*Xoo*) is one of the most destructive bacterial diseases on rice. Due to the resistance, toxicity and environmental issues of chemical bactericides, new biological strategies are still in need. Although peptaibols produced by *Trichoderma* spp. can inhibit the growth of several Gram-positive bacteria and plant fungal pathogens, it still remains unclear whether peptaibols have anti-*Xoo* activity to control bacterial leaf blight on rice. In this study, we evaluated the antibacterial effects of Trichokonins A (TKA), peptaibols produced by *Trichoderma longibrachiatum* SMF2, against *Xoo*. The *in vitro* antibacterial activity analysis showed that the growth of *Xoo* was significantly inhibited by TKA, with a minimum inhibitory concentration of 54 μg/mL and that the three TKs in TKA all had remarkable anti-*Xoo* activity. Further inhibitory mechanism analyses revealed that TKA treatments resulted in the damage of *Xoo* cell morphology and the release of intracellular substances, such as proteins and nucleic acids, from *Xoo* cells, suggesting the damage of the permeability of *Xoo* cell membrane by TKA. Pathogenicity analyses showed that the lesion length on rice leaf was significantly reduced by 82.2% when treated with 27 μg/mL TKA. This study represents the first report of the antibacterial activity of peptaibols against a Gram-negative bacterium. Thus, TKA can be of a promising agent in controlling bacterial leaf blight on rice.

## Introduction

Rice (*Oryza sativa*) is one of the most important staple food crops, serving for more than half of the population in the world (Hutin et al., 2016; Laborte et al., 2017). However, its production is severely affected by plant diseases caused by bacteria, fungi, viruses, nematodes and insects. Bacterial leaf blight caused by *Xanthomonas oryzae* pv. *oryzae* (*Xoo*) is one of the most destructive bacterial diseases on rice, which is prevalent in southeast Asia, west Africa, USA and northern Australia, resulting in up to 50% losses of rice production and representing a threat for food security (Niño-Liu et al., 2006; Quibod et al., 2020). *Xoo*, a rod-shaped Gram-negative bacterium, infects any growth stage of rice through hydathodes or wound sites on leaves and then colonizes in the space of epidermis to utilize the nutritional sources, leading to rice tissue necrosis and wilting (González et al., 2012; Ji et al., 2016; Timilsina et al., 2020).

Various management strategies have been used to minimize the loss of rice production caused by bacterial leaf blight. Chemical bactericides, including thiodiazole copper, thiazole zinc, phenazine-1-carboxamide, niclosamide 1,2,3,4-tetrahydro - β-carboline, S-thiazol-2-yl-furan-2-carbothioate, benzothiadiazole and bismerthiazol, were commonly used to control this disease (Shanmugaiah et al., 2010; Fan et al., 2017; Liang et al., 2018; Sahu et al., 2018; Jiang et al., 2019; Liu et al., 2020). In recent years, nano-technological products were considered as alternative strategies to control this disease, such as ZnO, MgO and MnO_2_ nanoparticles (Ogunyemi et al., 2020). Moreover, metal nanoparticles biosynthesized with chitosan, *Trichoderma* spp. or *Bacillus cereus* SZT1 also exhibited remarkable anti-*Xoo* activity (Abdallah et al., 2020; Ahmed et al., 2020; Shobha et al., 2020). However, the overuse of chemical bactericides and metal nanoparticles has resulted in environment pollution, increased resistance of pathogens and potential toxin to animals and humans. Therefore, the environmentally friendly and low-toxicity biological strategies have been contemplated as one of the most effective strategies to replace chemical bactericides and metal nanoparticles (Raaijmakers and Mazzola, 2012). To date, some bacterial strains, including *Pesudomonas* spp., *Streptomyces* spp. and *Paenibacillus polymyxa*, have been used as biological control agents to control this disease. Antibiotics produced by these bacterial strains played key roles in controlling this disease, such as phenazine-1-carboxamide from *P. aeruginosa* MML2212, pyoverdine from *P. chlororaphis* YL-1, carbazomycin B from S. roseoverticillatus 63 and fusaricidins P from *P. polymyxa* Sx3 (Shanmugaiah et al., 2010; Abdallah et al., 2019; Liu et al., 2021; Shi et al., 2021). However, effective strategies to control this disease by using biological control fungus is still lacking.

*Trichoderma* spp. are important fungal biological control agents, frequently living in root, soil, rotten wood and other environments with highly opportunistic potential and adaptability (Druzhinina et al., 2011). Many *Trichoderma* strains, including *T. harzianum*, *T. atroviride* and *T. reesei*, were effective to control soil-borne diseases caused by plant fungal pathogens (Green et al., 1999; Martinez et al., 2008; Seidl et al., 2009). The antimicrobial secondary metabolites (SMs) from *Trichoderma* spp., such as anthraquinones, stigmasterol, koninginins, harzianopyridone, pyrone and peptaibols, played key roles in controlling plant fungal diseases (Schirmböck et al., 1994; Vinale et al., 2006; Khan et al., 2020). Peptaibols are linear peptide antibiotics containing 5 to 20 amino acid residues with an acetylated N-terminus, a C-terminal amino alcohol and a high content of α-amino isobutyric acid (Aib) (Wiest et al., 2002). Antimicrobial activity analysis has revealed that peptaibols, including Trichorzianines, Trichorzins, Harzianines, Tricholongins and Trichotoxins, could effectively inhibit the growth of fungi, Gram-positive bacteria, viruses and nematodes (Bertelsen et al., 2007; Tamandegani et al., 2020). However, no peptaibol has been reported to inhibit Gram-negative bacteria till now.

*Trichoderma longibrachiatum* SMF2 (*Tl*SMF2) has been reported to produce peptaibols designated as Trichokonins (TKs), including Trichokonins A (TKA) with 20 amino acid residues and Trichokonins B (TKB) with 12 amino acid residues (Xiao-Yan et al., 2006; Zhou et al., 2019). Genome sequencing and gene deletion analysis revealed that the two non-ribosomal peptide synthetase (NRPS) encoding genes, *tlx1* and *tlx2*, are responsible for the biosynthesis of TKA and TKB, respectively (Xie et al., 2015; Zhou et al., 2019). TKs displayed broad-spectrum antimicrobial activity against several Gram-positive bacteria and plant fungal pathogens, but not against the analyzed Gram-negative bacteria, including *P. aeruginosa*, *Ralstonia solanacearum*, *Erwinia carotovora* and *Escherichia coli* (Xiao-Yan et al., 2006). In addition, although TKs could induce the resistance of Chinese cabbage against the infection caused by the Gram-negative bacterium *Pectobacterium carotovorum* subsp. *carotovorum*, TKs showed no antibacterial activity against this pathogen *in vitro* (Li et al., 2014).

In this study, we reported the antibacterial activity of TKA produced by *Tl*SMF2 against the Gram-negative bacterium *Xoo*. We found that *Tl*SMF2 could significantly inhibit the growth of *Xoo*, but the *tlx1*-deletion mutant strain could not. The purified TKA and its three components all showed remarkable anti-*Xoo* activity. Investigation of the inhibitory mechanism showed that TKA treatments led to the damage of the cell morphology of *Xoo* and the release of intracellular substances, such as proteins and nucleic acids, suggesting the damage of the permeability of cell membrane by TKA. Moreover, the pathogenicity analysis indicated that TKA had significant effect on controlling bacterial leaf blight caused by *Xoo* on rice, suggesting that TKA has the potential to be developed as an effective bio-bactericide to control this disease.

## Materials and methods

### Strains and culture conditions

The strains used in this study were listed in Table 1. *Xoo* and GFP tagged *Xoo* were grown on nutrient agar medium (Bacto™ Peptone 5 g/L, Yeast Extract 1 g/L, Sucrose 10 g/L, Beef extract 3 g/L and agar 15 g/L) at 28°C, or in the nutrient broth medium (Qian et al., 2013). The strains of WT, Δ*tlx1*, Δ*tlx2* and Δ*tlx1*&*tlx2* were grown on potato dextrose agar medium (fresh potato 200 g/L, Glucose 20 g/L and 15 g/L) at 28°C, or in the potato dextrose broth medium (Xiao-Yan et al., 2006). The mutants of *Tl*SMF2, Δ*tlx1*, Δ*tlx2* and Δ*tlx1*&*tlx2* were previously constructed (Zhou et al., 2019). The GFP tagged *Xoo* was previously constructed (Zhang et al., 2019).

**TABLE 1:** Strains used in this study.

Strains	Function	References
*Tl*SMF2	Wild type strain	Xiao-Yan et al., 2006
Δ*tlx1*	The *tlx1* gene deletion mutant strain of *Tl*SMF2	Zhou et al., 2019
Δ*tlx2*	The *tlx2* gene deletion mutant strain of *Tl*SMF2	Zhou et al., 2019
Δ*tlx1*&*tlx2*	The *tlx1* and *tlx2* gene double deletion mutant strain of *Tl*SMF2	Zhou et al., 2019
*Xoo* PXO99^A^	Philippine race 6	Salzberg et al., 2008
*Xoo*-GFP	*Xoo* PXO99^A^ harboring plasmid pUFZ75 (GFP-tagged strain), Km^R^	Zhang et al., 2019

Km^R^, kanamycin resistance.

### Extraction of secondary metabolites produced by *Tl*SMF2 and purification of Trichokonins A and its components

In order to extract the SMs, 0.25 cm2 plate of the mycelium margin of the WT, Δ*tlx1*, Δ*tlx2* or Δ*tlx1*&*tlx2* strain of *Tl*SMF2 was grown on the plate containing 15 mL potato dextrose agar medium for 12 days, and then the potato dextrose agar medium was dipped into 200 mL ethanol for 24 h. The mixture was centrifuged at 10,000 g, and the supernatant was collected and was dried by using freeze-drying. The dried SMs were dissolved with 2 mL methanol, which were used for the analysis of the anti-*Xoo* activity.

TKA and Trichokonin VI (TK VI), Trichokonin VII (TK VII) and Trichokonin VIII (TK VIII) were purified and identified as previously described (Xiao-Yan et al., 2006; Zhou et al., 2019). Briefly, approximately 2 × 10^7^ spores of the WT strain were inoculated into 100 mL potato dextrose broth medium in a 500 mL flask, which were cultured at 28°C with shaking at 180 rpm for 12 days. Then the potato dextrose broth culture was centrifuged at 10,000 *g*, and the collected supernatant (40 mL) was loaded on a Cleanert C18 SPE Cartridge, and the TKA was eluted by 2 mL methanol. The eluted TKA was furtuer purified by using HPLC on a reversed phase analytical column (Shimadzu, Japan) that were eluted with methanol/ddH_2_O (84:16, v/v) at a flow rate of 1.0 mL/min. The chromatogram was monitored at 203 nm. TKs VI, VII and VIII were collected together as purified TKA, or collected separately as purified TKs VI (retention time 17.35 min), VII (retention time 20 min) and VIII (retention time 22.5 min) according to previous identification (Xiao-Yan et al., 2006). The purified TKA and each TKA component (TKs VI, VII or VIII) were dried by using freeze-drying. The purified TKA and each component were dissolved in methanol at a concentration of 10 mg/mL as stock solution. The stock solution was filter-sterilized (0.22 μm) and then used for the analysis of their anti-*Xoo* activities.

### Analysis of the anti-*Xoo* activity

The anti-*Xoo* activity of *Tl*SMF2 was analyzed by using the method described previously by Dos et al. with some modification (Dos Santos et al., 2015). Briefly, 0.25 cm^2^ plate of the mycelium margin of the WT, Δ*tlx1*, Δ*tlx2* or Δ*tlx1*&*tlx2* was transferred to the center of a test plate containing 15 mL nutrient agar medium or to a 250 mL flask containing 50 mL nutrient broth medium, both of which contained *Xoo* at 1 × 10^7^ CFU/mL. The co-cultures were incubated at 28°C with (for nutrient broth medium) or without (for nutrient agar medium) shaking at 180 rpm for 2 to 6 days. The antagonistic circle around the colony of *Tl*SMF2 or its mutants on the test plate was observed, and the OD_600_ of the lens cleaning tissue filtered liquid culture was recorded.

The anti-*Xoo* activities of SMs, TKA and TKs VI, VII and VIII were analyzed using agar well-diffusion assay as described previously with some modification (Nanda and Saravanan, 2009). Briefly, *Xoo* was cultured in nutrient broth medium to 1 × 10^7^ CFU/mL, and 200 μL suspension of *Xoo* was spread on nutrient agar medium in a test plate containing 15 mL nutrient agar medium by using a sterile triangular glass coating rod. Then, the wells of 5 mm diameter were loaded on the surface of the test plate and the extracted SMs (20 μL, 40 μL, 60 μL, and 80 μL), 80 μg of TKA and TKs VI, VII and VIII were poured into the wells. The test plate was incubated at 28°C for 3 days, and the diameter of the inhibition zone was recorded. Moreover, 5 × 10^8^ *Xoo* cells were inoculated into 250 mL flask containing 50 mL nutrient broth medium containing the extracted SMs (0.2%, 0.4%, 0.6%, and 0.8%, v/v), and were incubated with shaking (180 rpm) at 28°C for 48 h, and the OD_600_ of the culture was recorded every 2 h. Methanol (0.8%, v/v) was used as the negative control. Three replicates were performed in each treatment, and the experiment was repeated three times.

### Determination of minimum inhibitory concentration

The MICs of TKA and TKs VI, VII and VIII against *Xoo* were determined using the method as described previously with some modification (Du et al., 2020). Briefly, 2 × 10^6^ *Xoo* cells were inoculated into the column of a 96-well plate containing 200 μL nutrient broth medium, and different concentration (0 to 100 μg/mL) of TKA or TKs VI, VII or VIII was added to the 200 μL nutrient broth medium. The 96-well plate was incubated at 28°C for 2 days. The OD_600_ of the culture was recorded every 2 h using Bioscreen C optical growth analyzer (Bioscreen, Finland) to assess the growth of *Xoo*. The lowest concentration of TKA or TKs VI, VII or VIII that completely inhibited the growth of *Xoo* was regarded as the MIC. The results were further confirmed by repeating the experiment in 30 mL bottles containing with 8 × 10^7^ *Xoo* cells in 8 mL nutrient broth medium with different concentrations (0 to 100 μg/mL) of TKA or TKs VI, VII or VIII at 28°C for 2 days. Methanol (1.2%, v/v) was used as the negative control. Three replicates were performed in each treatment, and the experiment was repeated three times.

### Transmission electron microscopy observation of *Xoo*

The cell morphology of *Xoo* was observed using transmission electron microscopy as described previously with some modification (Erdmann et al., 2017). *Xoo* was cultured in nutrient broth medium to 1 × 10^9^ CFU/mL. The cells were collected by centrifugation at 6,000 *g* for 5 min, and then were washed with sterile ddH_2_O for three times. The cells were suspended in sterile ddH_2_O, and were treated with 54 μg/mL TKA at 28°C for 24 h. After treatment, 5 μL cell preparation was adsorbed onto the carbon-coated copper grids for 1.5 min, and then the cells were stained with 2% uranyl acetate for 30 s. Images were pictured by using transmission electron microscopy (JEOL, Japan). Methanol (0.3%, v/v) treatment was used as the negative control. At least 50 *Xoo* cells were detected in each treatment. Three replicates were performed in each treatment, and the experiment was repeated three times.

### Atomic force microscopy observation of *Xoo*

The cell morphology of *Xoo* was observed using atomic force microscopy with a previously described method with some modification (Tang et al., 2020). Cell preparations with 1 × 10^9^ CFU/mL were obtained as described in the method of TEM observation. After treated with 54 μg/mL TKA at 28°C for 24 h, 2.5 μL cell preparation was deposited onto freshly cleaved mica and was dried in a chamber at room temperature. Atomic force microscopy was performed using a Multimode VIII AFM with Nanoscope V controller (Bruker AXS, Germany), and images were pictured in the scanasyst mode under air condition. Methanol (0.3%, v/v) treatment was used as the negative control. At least 50 *Xoo* cells were detected in each treatment. Three replicates were performed in each treatment, and the experiment was repeated three times.

### Detection of the release of intracellular substances from *Xoo* cells treated by Trichokonins A

The method to detect the release of intracellular substances from cells was carried out as previously described with some modification (Liang et al., 2020). Cell preparations with 1 × 10^9^ CFU/mL were obtained as described in the method of TEM observation. After treated with 54 μg/mL TKA at 28°C for 24 h, the OD_600_ of the culture was recorded, and the supernatant was collected by centrifugation at 8,000 *g* for 5 min. The concentration of nucleic acids in the supernatant was measured by recording the absorbance of the supernatant at 260 nm using NanoDrop™ One (Thermo Scientific, USA). The supernatant was concentrated (1:20) by using a 3, 000 Da ultrafilter tube (Merck Millipore, USA), and then the concentration of protein in the concentrated supernatant was measured by using Pierce BCA Protein Assay Kit (Thermo Scientific, USA) with bovine serum albumin as the standard.

The GFP tagged *Xoo* cells were prepared and treated with TKA using the same method as WT *Xoo.* The concentration of released GFP protein in the supernatant of GFP tagged *Xoo* cells was detected by Western blot using the method described previously with some modification (Shi et al., 2019). Proteins were separated by SDS-PAGE at 90 V for 30 min, and then at 120 V for 90 min. Proteins in the gel were transferred onto PVDF membrane at 120 mA for 60 min. The PVDF membrane was incubated in the blocking buffer for 1 h, and then in a new blocking buffer containing the primary antibody (GFP-Tag Mouse mAb, 1: 5000, Abmart, China) for 2 h, followed by an incubation in a new blocking buffer containing the secondary antibody (Goat Anti-Rabbit$Mouse IgG-HRP, 1: 5000, Abmart, China) for 1 h. After that, the PVDF membrane was stained with the Super ECL Plus kit and imaged using FluorChem M (Alpha Innotech, USA). Methanol (0.3%, v/v) treatment was used as the negative control. Three replicates were performed in each treatment, and the experiment was repeated three times.

### Pathogenicity analysis

The pathogenicity of *Xoo* on rice was analyzed with the method as described previously with some modification (Zhang et al., 2019). Briefly, the susceptible rice cultivar IR24 was planted in greenhouse under a cycle of light at 28°C for16 h and dark at 25°C for 8 h. The 4 weeks old rice seedlings were dipped into water containing TKA at the concentration of 13.5 μg/mL, 27 μg/mL or 54 μg/mL for 2 days. After that, the rice leaves were inoculated with *Xoo* cells suspension in sterile distilled water (OD_600_ = 0.5) by the method of leaf-clipping. Moreover, TKA at the concentration of 13.5 μg/mL, 27 μg/mL or 54 μg/mL also was sprayed to the *Xoo*-inoculated rice leaves on the 5*^th^* and the 10*^th^* day. Lesion lengths on the rice leaves were measured after 14 days from the inoculation. Methanol (0.3%, v/v) was used as the negative control. At least 50 rice leaves were inoculated in each treatment. Three replicates were performed in each treatment, and the experiment was repeated three times.

### Data analysis

All analysis was conducted by using SPSS 14.0 (SPSS Inc., Chicago, IL, USA). Significant differences were determined *via* the hypothesis test of percentages (*t*-test) (** *p* < 0.01).

## Results

### *Tl*SMF2 and its secondary metabolites exhibited anti-*Xoo* bacterial activity

**FIGURE 1:**
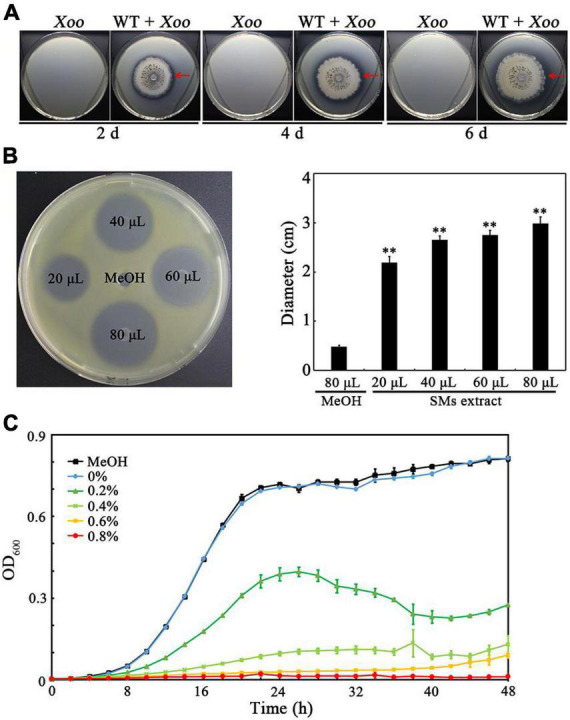
Determination of the anti-*Xoo* activities of *Tl*SMF2 and its SMs. **(A)** The representative anti-*Xoo* activity of *Tl*SMF2 on the test plates containing nutrient agar medium after co-cultured for 2, 4 and 6 days. **(B)** The representative *Xoo*-inhibition zones of the SMs extracted from *Tl*SMF2 on the test plate containing nutrient agar medium (left) and the diameters of the zones (right). The diameters were data from three repeats (mean ± S.D.). Asterisk indicates significant difference compared with the control of MeOH (** means *P* < 0.01). **(C)** The growth of *Xoo* in nutrient broth medium containing different volume of the SMs (0%, 0.2%, 0.4%, 0.6%, and 0.8%, v/v) extracted from *Tl*SMF2. MeOH, the nutrient broth medium containing methanol (0.8%, v/v). The graph shows data from triplicate experiments.

To investigate whether *Tl*SMF2 has anti-*Xoo* activity, they were co-cultured on nutrient agar medium. An antagonistic circle could be clearly observed around the margin of *Tl*SMF2 after co-cultured for 2 to 6 days (Figure 1A), indicating that *Tl*SMF2 likely produced SMs to inhibit the growth of *Xoo*. Then, the SMs produced by *Tl*SMF2 were extracted and the anti-*Xoo* activity of the SMs extract was analyzed. Compared with the negative control of methanol, inhibition zones could be clearly seen on the test plate and their diameter ranged from 2.19 to 2.99 cm when *Xoo* was treated with the SMs extract from 20 to 80 μL (Figure 1B). Correspondingly, the growth of *Xoo* in nutrient broth liquid medium was severely inhibited by the addition of 0.2 to 0.6% (v/v) SMs extract, and almost complete inhibited by the addition of 0.8% (v/v) (Figure 1C). These results indicated that one or more SMs produced by *Tl*SMF2 had anti-*Xoo* activity.

### The anti-*Xoo* activity of *Tl*SMF2 was attributed to Trichokonins A

**FIGURE 2:**
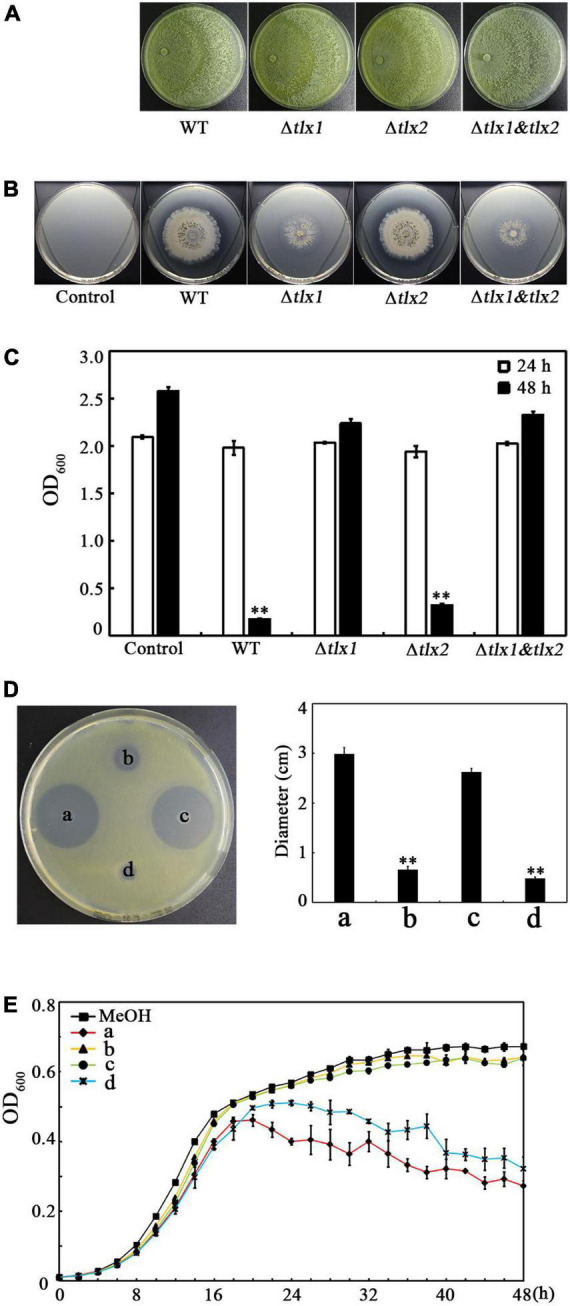
Effect of the gene *tlx1* and *tlx2* deletion on the anti-*Xoo* activity of *Tl*SMF2. **(A)** Growth of the WT *Tl*SMF2 and its mutants on the test plates containing potato dextrose agar medium after cultured for 4 days. The picture shows a representative of three repeats. **(B)** The representative anti-*Xoo* activities of the WT *Tl*SMF2 and its mutants on the test plates containing nutrient agar medium after co-cultured for 6 days. **(C)** The OD_600_ of the co-cultures with the WT *Tl*SMF2 and its mutants after 24 and 48 h. Control: *Xoo* cultured without WT *Tl*SMF2 or its mutants. WT, the co-culture of *Xoo* with WT *Tl*SMF2. Δ*tlx1*, Δ*tlx2* and Δ*tlx1*&*tlx2*, the co-cultures of *Xoo* with the mutant strains Δ*tlx1*, Δ*tlx2* and Δ*tlx1*& *tlx2*, respectively. **(D)** The representative *Xoo*-inhibition zones of the SMs extracted from WT *Tl*SMF2 and its mutants on the test plate containing nutrient agar medium (left) and the diameters of the zones (right). a, b, c and d represent the SMs produced by strains WT *Tl*SMF2 (a), Δ*tlx1*(b), Δ*tlx2* (c) and Δ*tlx1*&*tlx2* (d), respectively. The diameters were data from three repeats (mean ± S.D.). Asterisk indicates significant difference compared with the control of MeOH (** means *P* < 0.01). **(E)** The growth of *Xoo* in nutrient broth medium containing the SMs (0.8%, v/v) extracted from the WT *Tl*SMF2 and its mutants. MeOH, the nutrient broth medium containing methanol (0.8%, v/v). The graphs show data from triplicate experiments.

Because TKs have been shown to be a kind of antimicrobial peptides in the SMs produced by *Tl*SMF2 (Xiao-Yan et al., 2006), we speculated that the TKs produced by *Tl*SMF2, TKA and/or TKB, may have anti-*Xoo* activity. To test this hypothesis, we analyzed the anti-*Xoo* activities of the wild-type strain of *Tl*SMF2 and its gene-deletion strains Δ*tlx1*, Δ*tlx2*, and Δ *tlx1*&*tlx2* previously constructed (Zhou et al., 2019). On potato dextrose agar plates, the three mutant strains displayed similar growth rate to wild-type *Tl*SMF2 (Figure 2A), suggesting that gene deletion had little impact on the growth of these mutants. On the co-culture plates containing nutrient agar medium, Δ*tlx2* formed an antagonistic circle with a size similar to that of WT, but Δ*tlx1* and Δ*tlx1*& *tlx2* both formed a negligible one, indicating that Δ*tlx1* and Δ*tlx1*& *tlx2* almost completely lost the anti-*Xoo* activity, but Δ*tlx2* still retained this activity (Figure 2B). This was further supported by co-culture in nutrient broth liquid medium. After 48 h co-culture of *Xoo* with WT or its mutants, the OD_600_ of the control (containing only *Xoo*) and the co-cultures of *Xoo* with Δ*tlx1* or Δ*tlx1*&*tlx2* all reached to approximately 2.5, but those of the co-cultures of *Xoo* with WT and Δ*tlx2* were only 0.18 and 0.32, respectively (Figure 2C), indicating that the growth of *Xoo* was significantly inhibited by WT or Δ*tlx2*, but not by Δ*tlx1* or Δ*tlx1*&*tlx2.* Because gene *tlx1* encodes TKA and gene *tlx2* encodes TKB in *Tl*SMF2 (Zhou et al., 2019), these results suggested that the anti-*Xoo* activity of *Tl*SMF2 was mainly attributed to the production of TKA in its SMs. This was also supported by analyzing the anti-*Xoo* activities of the SMs from WT and its mutants in solid and liquid culture. On the test plate containing nutrient agar medium, the SMs from WT and Δ*tlx2* showed noticeable inhibition zones, but those from Δ*tlx1* or Δ*tlx1*&*tlx2* did not (Figure 2D). In nutrient broth liquid culture, the SMs from WT and Δ*tlx2* both showed a noticeable inhibitory effect on the growth of *Xoo*, but those from Δ*tlx1* or Δ*tlx1*&*tlx2* did not (Figure 2E).

**FIGURE 3:**
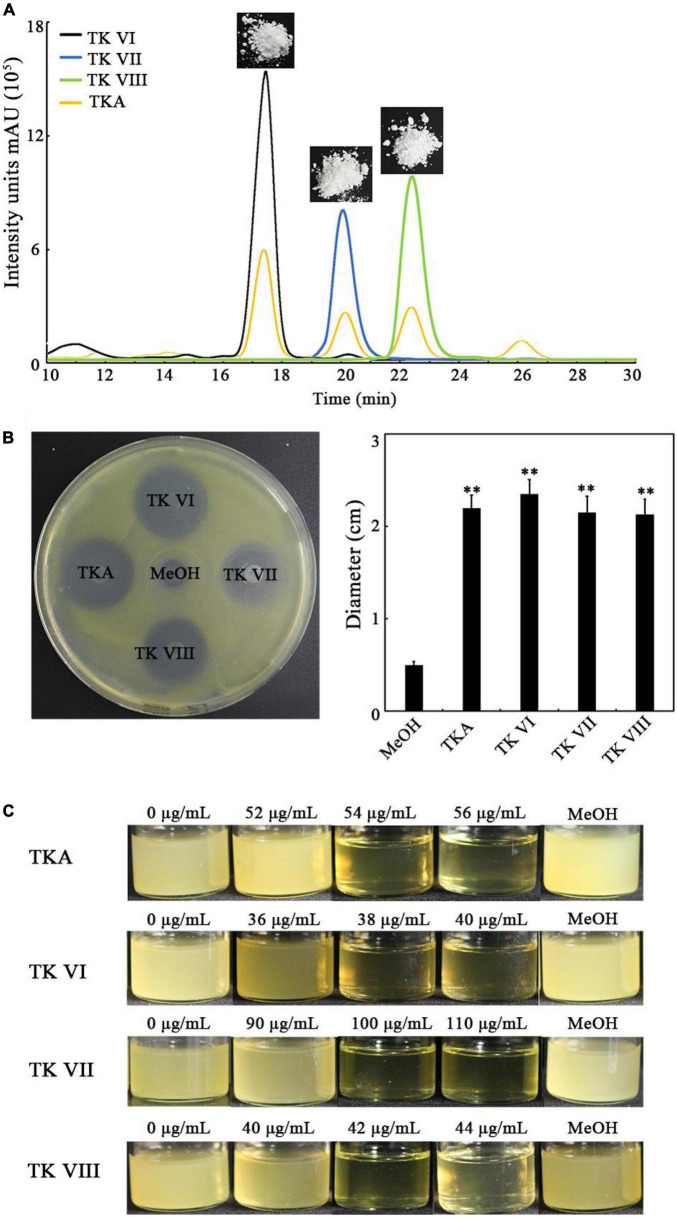
Determination of the anti-*Xoo* activities of TKA and TKs VI, VII and VIII. **(A)** The representative purified TKA and TKs VI, VII and VIII detected by using HPLC. **(B)** The representative *Xoo*-inhibition zones of 80 μg TKA and TKs VI, VII and VIII on the test plate containing nutrient agar medium (left) and the diameters of the zones (right). The diameters were data from triplicate experiments (mean ± S.D.). Asterisk indicates significant difference compared with the control of MeOH (** means *P* < 0.01). **(C)** The growth of *Xoo* in 8 mL nutrient broth medium containing different concentrations (0 to 110 μg/mL) of TKA or TKs VI, VII or VIII, respectively. MeOH, the nutrient broth medium containing methanol (1.2%, v/v).

To confirm the anti-*Xoo* activity of TKA, we purified TKA (Figure 3A) and analyzed its anti-*Xoo* activity. The purified TKA showed remarkable anti-*Xoo* activity on the test plate (Figure 3B). It has been reported the TKA produced by *Tl*SMF2 contained three TKs, that is, TKs VI, VII and VIII (Xiao-Yan et al., 2006). We then purified the three TKs of TKA separately (Figure 3A) and tested their anti-*Xoo* activities. As shown in Figure 3B, the three TKs all showed remarkable anti-*Xoo* activity. To further compare their anti-*Xoo* activities, we determined the MIC of TKA and TKs VI, VII and VIII against *Xoo* by monitoring their inhibitory effects on the growth of *Xoo* under different concentrations. The MIC of TKA against *Xoo* was 54 μg/mL. Compared with TKA, both TKs VI and VIII showed a stronger anti-*Xoo* activity, with the MICs at 38 μg/mL and 42 μg/mL, respectively, but the anti-*Xoo* activity of TK VII was much weaker, with the MIC at 100 μg/mL (Figure 3C). Thus, TK VI had the strongest anti-*Xoo* activity among the three components of TKA.

### Trichokonins A treatment led to the damage of the *Xoo* cell morphology and the release of intracellular substances

**FIGURE 4:**
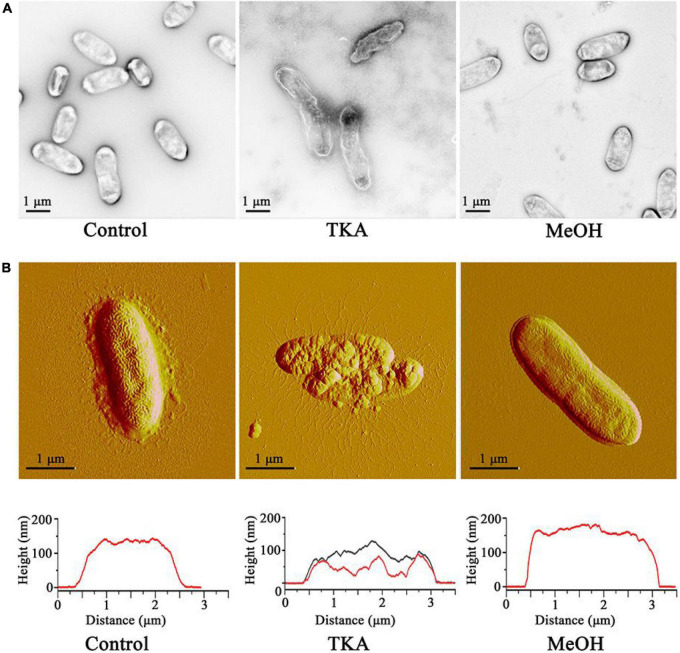
Effect of TKA on the cell morphology of *Xoo*. **(A)** Morphology of representative *Xoo* cells observed by using transmission electron microscopy. **(B)** Morphology (up) and height (down) of representative *Xoo* cells observed by using atomic force microscopy. Control, a representative of *Xoo* cells untreated with methanol or TKA. TKA, two representative of *Xoo* cells treated with 54 μg/mL TKA for 24 h. MeOH, a representative of *Xoo* cells treated with methanol (0.3%, v/v) for 24 h. Fifty *Xoo* cells were observed in each treatment. Each treatment was repeated three times.

Cell morphology is fundamental for the cellular functions (Fukuda et al., 2021). To investigate whether TKA affects the cell morphology of *Xoo, Xoo* cells treated with TKA or methanol (as a negative control) was observed by using transmission electron microscopy and atomic force microscopy. The result of transmission electron microscopy observation showed that, after treated with 54 μg/mL TKA for 24 h, 96% *Xoo* cells displayed distorted and irregular morphology, with separated cell membrane from the cell envelop and intracellular permeated uranyl acetate, suggesting the damage of the cell membrane permeability. In contrast, the cells treated with methanol all displayed intact, normal morphology, with uranyl acetate being attached on the cell surface (Figure 4A). Correspondingly, the atomic force microscopy observation showed that the *Xoo* cells untreated and those treated with methanol displayed similar intact and smooth surfaces and height, but those treated with 54 μg/mL TKA for 24 h were clearly destroyed, with roughed surfaces and significantly decreased height (Figure 4B). In addition, it seemed that intracellular substances were released from the *Xoo* cells treated with 54 μg/mL TKA based on the atomic force microscopy observation (Figure 4B).

**FIGURE 5:**
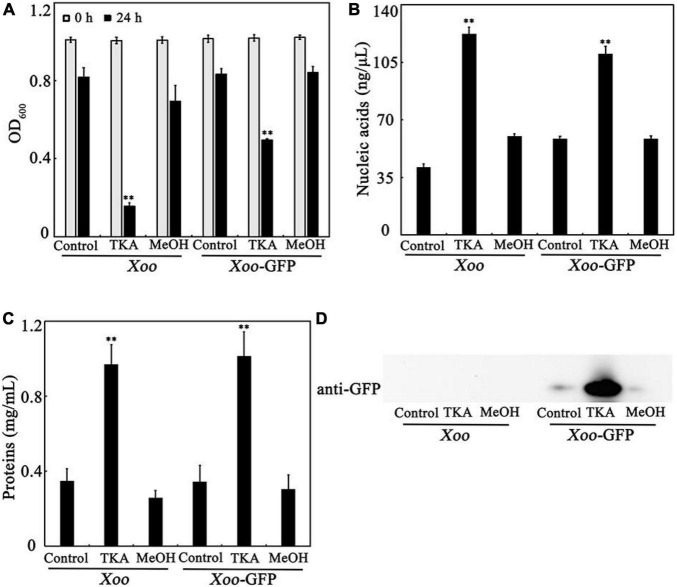
Effect of TKA on the release of intracellular substances from *Xoo* cells. **(A)** The OD_600_ of the cultures of WT *Xoo* and GFP tagged *Xoo* (*Xoo*-GFP) in ddH_2_O incubated at 28°C with or without TKA for 0 and 24 h. **(B)** The concentration of nucleic acids in the supernatants of the cultures of WT *Xoo* and GFP tagged *Xoo* incubated with or without TKA for 24 h. **(C)** The concentration of total proteins in the supernatants of the cultures of WT *Xoo* and GFP tagged *Xoo* incubated with or without TKA for 24 h. **(D)** Western blot detection of the GFP protein in the supernatants of the cultures of WT *Xoo* and GFP tagged *Xoo* incubated with or without TKA for 24 h. Control, WT *Xoo* and GFP tagged *Xoo* incubated without methanol or TKA. TKA, WT *Xoo* and GFP tagged *Xoo* incubated with 54 μg/mL TKA. MeOH, WT *Xoo* and GFP tagged *Xoo* incubated with methanol (0.3%, v/v). The graphs show data from triplicate experiments (mean ± S.D.). The picture in D is a representative of three repeats. Asterisk indicates significant difference compared with the control (** means *P* < 0.01).

To confirm the effects of TKA treatment on the intracellular substances, *Xoo* cells were treated with 54 μg/mL TKA for 24 h, and then the cell density (OD_600_) and the concentrations of nucleic acids and proteins in the supernatant were determined and compared to those untreated or treated with methanol. After TKA treatment, the OD_600_ of the cell suspension reduced 84.4%, but that treated with methanol reduced only 19.2%, similar to that untreated with TKA or methanol (Figure 5A), which indicated the damage of *Xoo* cells by TKA. The concentration of both nucleic acids and proteins in the supernatant of *Xoo* cells treated with TKA were significantly higher (approximately 3 folds and 5 folds, respectively) than those in the supernatant of *Xoo* cells untreated or treated with methanol (Figures 5B,C), suggesting that TKA treatment led to much more release of intracellular nucleic acids and proteins from *Xoo* cells. To further confirm the effects of TKA, the endogenously expressed GFP protein was used as an indicator to investigate the leakage of intracellular substances. Similar to the case in WT *Xoo*, TKA treatment led to significant decrease in the cell number of GFP tagged *Xoo* and significant increase in the release of intracellular nucleic acids and proteins (Figures 5A–C). Moreover, a large concentration of GFP protein was detected by western blot in the supernatant of the GFP tagged *Xoo* cells treated with TKA for 24 h, but only trace concentration of GFP protein was detected in the supernatant of the GFP tagged *Xoo* cells untreated or treated with methanol (Figure 5D), indicating the severe leakage of intracellular GFP protein from *Xoo* cells treated with TKA. Altogether, these results demonstrated that TKA treatment led to the severe release of intracellular substances from *Xoo* cells, suggesting that the permeability of the cell membrane of *Xoo* was likely destroyed by TKA.

### Trichokonins A reduced the pathogenicity of *Xoo* on rice

**FIGURE 6:**
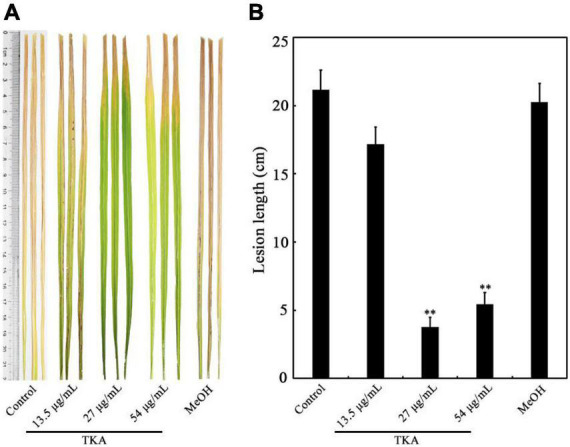
Effect of TKA on the pathogenicity of *Xoo* on rice. **(A)** The representative lesion lengths on the leaves of the *Xoo*-inoculated rice seedlings of cultivar IR24 treated with different concentration of TKA (13.5 μg/mL, 27 μg/mL and 54 μg/mL) in greenhouse for 14 days. At least 50 rice leaves were recorded in each treatment. Each treatment was repeated three times. **(B)** The lesion lengths on the leaves of the *Xoo*-inoculated rice seedlings of cultivar IR24 treated with different concentration of TKA (13.5 μg/mL, 27 μg/mL and 54 μg/mL) in greenhouse for 14 days. The graph shows data from triplicate experiments (mean ± S.D.). Asterisk indicates significant difference compared with the control of MeOH (** means *P* < 0.01). Control, the *Xoo*-inoculated rice seedling leaves of cultivar IR24 untreated with methanol or TKA. MeOH, the *Xoo*-inoculated rice seedling leaves of cultivar IR24 treated with methanol (0.3%, v/v).

Because TKA showed remarkable anti-*Xoo* activity, the role of TKA in controlling bacterial leaf blight on rice caused by *Xoo* was further evaluated by pathogenicity analysis. The lesion length on the leaf of rice untreated with TKA or methanol was approximately 21.2 cm, which was approximately 20.3 cm on that treated with methanol. In contrast, when the rice was treated with 13.5 μg/mL, 27 μg/mL and 54 μg/mL TKA, the lesion length on rice leaf was reduced to approximately 17.2 cm, 3.8 cm and 5.4 cm, respectively. The calculated protective efficiency was approximately 18.8%, 82.2% and 74.3% when the rice was treated with 13.5 μg/mL, 27 μg/mL and 54 μg/mL TKA, respectively (Figure 6). This result showed that TKA could reduce the pathogenicity of *Xoo* on rice, and that 27 μg/mL TKA had the best protective efficiency.

## Discussion

Peptaibols are a class of linear peptides mainly produced by *Trichoderma* and *Emericellopsis* (Daniel and Filho, 2007). Studies have shown that peptaibols exhibited broad-spectrum antimicrobial activity against several Gram-positive bacteria and plant fungal pathogens. For example, TKs exhibited broad-spectrum antimicrobial activity against *B. subtilis*, *S. aureus* and *Fusarium oxysporum* (Xiao-Yan et al., 2006). Trichotoxins showed antibacterial activity against *B. stearothermophilus* (Chutrakul et al., 2008). Emericellipsin A showed significant inhibitory activity against *Aspergillus niger* ATCC 16404 and *Candida albicans* ATCC 14053 (Kuvarina et al., 2021). A recent study found that water-soluble trichogin GA IV-derived peptaibols inhibited the growth of plant fungal pathogens, such as *Botrytis cinerea*, *F. graminearum*, *Penicillium expansum* and *Pyricularia oryzae* (Baccelli et al., 2022). However, there has been no peptaibol being shown to have antibacterial activity against Gram-negative bacteria. In this study, we found that TKA from *Tl*SMF2 showed significant antibacterial activity against the Gram-negative bacterium *Xoo*. Moreover, the three Trichokonins in TKA, including TKs VI, VII and VIII, all showed remarkable anti-*Xoo* activity. Notably, the MIC of TK VII against *Xoo* was much higher than those of TKs VI and VIII, which may be caused by the amino acid difference in their sequences. The 17*^th^* amino acid residue is Iva in TK VII, but is Aib in TK VI and VIII. Further study needs to be conducted to decipher this difference. In addition, whether TKA has antibacterial activity against other Gram-negative bacterial pathogens awaits further investigation.

Most peptaibols are membrane-active compounds with the ability to form multimeric ion channels in lipid bilayer membranes (Chugh et al., 2002; Marik et al., 2019). This is considered to be the main antimicrobial mechanism of peptaibols. The pore formation in the membranes eventually results in the leakage of intracellular substances (Milov et al., 2016). For example, emericellipsin A disrupted the cells membrane of *Staphylococcus aureus*, resulting in the influx of propidium iodide into cells (Rogozhin et al., 2018). In our previous studies, TK VI could induce the autophagy through an influx of Ca^2+^ to inhibit the growth of HepG2 cancer cells (Shi et al., 2012). Moreover, TK VI could change the cell morphology of plant fungal pathogen *F. oxysporum* and induce the production of reactive oxygen species to inhibit its growth (Shi et al., 2010). In this study, consistent with the effect of emericellipsin A, we found the application of TKA led to clearly changed cell morphology of *Xoo* and significant release of intracellular substances from *Xoo* cells, such as nucleic acids and proteins. Thus, TKA likely adopts the similar antimicrobial strategy against *Xoo* as other peptaibols against Gram-positive bacteria, fungi and mammal cells, which rupture the integrity of *Xoo* cell membrane and promotes the cell leakage, thereby leading to the morphology change and the cell death.

In our previous study, we found that TKs from *Tl*SMF2 controlled the tobacco mosaic virus by increasing the production of reactive oxygen species and phenolic compounds, as well as enhancing the expression of pathogenesis-related genes (Luo et al., 2010). Moreover, TKs from *Tl*SMF2 also could enhance the resistance of Chinese cabbage against the infection of pathogen through inducing the production of reactive oxygen species and the expression of pathogenesis-related genes (Li et al., 2014). In addition, Viterbo et al. (2007) found that the application of two synthetic 18-amino-acid peptaibol isoforms (TvBI and TvBII) from *T. virens* strain Gv29-8 induced the expression of plant resistance related enzymes, including hydroxyperoxide lyase, phenylalanine ammonia lyase and peroxidase, to against the infection of *P. syringae* pv. *lachrymans* on cucumber seedlings. Therefore, the induced plant resistance against pathogens also plays an important role in the control of plant diseases by peptaibols. In this study, we found the pathogenicity of *Xoo* on rice significantly reduced after the application of TKA at the concentration of 27 μg/ml, only half of the MIC of TKA against *Xoo*. It would be an interesting topic for the future investigation to understand the underlined molecular mechanisms of TKA eliciting resistance response in rice. In recent years, some microbial SMs have been used to against *Xoo* and to decrease the incidence of bacterial leaf blight on rice. For example, staurosporine produced by *Streptomyces* sp. MJM4426 inhibited the growth of *Xoo* with the MIC at 200 μg/mL (Cheng et al., 2016). Difficidin and bacilysin produced by *B. amyloliquefaciens* FZB42 also exhibited anti-*Xoo* activity, and the protective rates to bacterial leaf blight reached to 58.82% and 72.31% with the concentration at 50 μg/mL, respectively (Wu et al., 2015). Decyl alcohol and 3,5,5-trimethylhexanol produced by *Bacillus* strain D13 inhibited the growth of *Xoo* with the MIC at 480 μg/mL and 2.4 mg/mL, respectively (Xie et al., 2018). In this study, we found the MIC of TKA produced by *Tl*SMF2 against *Xoo* was 54 μg/mL, and that 82.2% protective rate could be achieved with TKA at the concentration of 27 μg/mL. However, the protective rate decreased to 74.3% at the concentration of 54 μg/mL, suggesting that the excess of TKA may be toxic to rice. The high protective rate indicates that TKA can be of a promising agent in controlling bacterial leaf blight on rice.

## Data availability statement

The original contributions presented in this study are included in the article/supplementary material, further inquiries can be directed to the corresponding authors.

## Author contributions

X-YS and H-YC: conceptualization. H-YL: extraction of SMs. Kun-Liu: purification of TKs. M-LS: TEM observation. H-NS: AEM observation. Y-QZ: analysis of anti-*Xoo* activity and pathogenicity and writing – original draft preparation. SZ, X-LC, and Y-ZZ: writing – review and editing. All authors have read and agreed to the published version of the manuscript.
